# Omics and Multi-Omics Insights into Plant Responses to Abiotic Stresses

**DOI:** 10.3390/plants15050799

**Published:** 2026-03-05

**Authors:** Rudo Ngara, Yuri Shavrukov

**Affiliations:** 1 Department of Plant Sciences, University of the Free State, Qwaqwa Campus, Phuthaditjhaba 9866, South Africa; 2 College of Science and Engineering, Biological Sciences, Flinders University, Adelaide, SA 5042, Australia

Plants are often exposed to abiotic stresses such as drought, salinity, heat, cold, freezing, nutrient deficiency, and heavy metal toxicity, which limit their growth and development. In the case of crops, abiotic stresses may reduce yields, leading to insufficient food supply for the growing population. In addition, these stresses may occur concomitantly under field conditions, resulting in diverse effects on plant growth and metabolism. Whereas stress combinations such as drought and ozone may result in potentially positive interactions, others, including drought and heat or salinity with nutrient deficiency, may cause extensive plant cell damage and greater yield reductions than the individual stresses alone. Furthermore, the frequency and magnitude of stresses, such as drought and heat, will likely increase with global warming. Consequently, research interest in understanding the complex responses of plants to individual and combinations of abiotic stresses is increasing.

Omics technologies are also improving our insights into stress-responsive changes in the transcriptome, proteome, and metabolome of plants, while the sequencing of entire plant genomes is providing resources to guide crop improvement strategies. Other research groups are working on validating the roles of these ‘omics-derived data’ in stress adaptation using various methodologies, including transgenic plants, seed priming, and exogenous application of growth-promoting compounds.

The Special Issue “Omics and Multi-Omics Insights into Plant Responses to Abiotic Stresses” consists of 13 papers that broaden our insights into plant responses to individual abiotic stresses and their combinations using various omics approaches, including the functional validation of such data. Therefore, this Special Issue expands our understanding of plant tolerance to various abiotic stresses through omics and multi-omics approaches.

The perennial submerged aquatic plant species, *Potamogeton wrightii*, is widely distributed across China and used for medicine and as a highly nutritional feed for animals but this species is sensitive to phosphorus stress. Homeostasis of P is essential for optimal plant growth and development, with highly coordinated signaling mechanisms regulating phosphorus deficiency and excess. The authors report leaf chlorosis and chlorophyll loss in response to high phosphorus treatment, as well as increased MDA and H_2_O_2_ contents and higher POD, SOD, and CAT activities. The RNA-seq approach used in the study showed a thousand differentially expressed genes common across all phosphorus treatments in *P. wrightii* plants. Using an UPLCMS/MS system, 3408 metabolites arranged into 21 categories were detected in leaves. Combined transcriptomic and metabolomic analysis revealed six differentially accumulated metabolites (DAMs) and 19 differentially expressed genes (DEGs) showing down- and upregulation in glycerolipid and glycerophospholipid metabolism in response to low and high phosphorus stress in *P. wrightii* plants. The presented report is an important step towards understanding the mechanisms underlying plant responses to phosphorus stress [1].

Fungal endophytes are healthy, asymptomatic fungi living inside plants, and they are the focus of a specific area of plant phytopathology and mycology. The selected endophyte EF0801 was identified as being similar to *Sordariomycetes*, and it was used for the treatment of rice seedlings. The authors studied the benefit of endophyte infection in rice seedlings grown in a solution with sodium carbonate, 10 mM Na_2_CO_3_, which resulted in increased plant height but reduced root length. The application of gas chromatography and mass spectrometry revealed the enrichment of starch and sucrose metabolism, together with three amino acids (alanine, aspartate, and glutamate) in the infected rice seedlings grown under carbonate treatment compared with control plants. Together with the RNA-seq technique used, the authors found six DEGs and six DAMs involved in the response of the rice plants to Na_2_CO_3_ treatment. One of the most important genes identified was hexokinase, *HXK*, which encodes an enzyme that converts glucose and fructose in their respective metabolite pathways. The results of the analysis of the transcriptome and metabolome in the rice seedlings infected with the endophyte fungus provide knowledge and wide perspectives for improving rice plant tolerance to carbonate and alkaline soils [2].

An opinion paper discusses the use of polyethylene glycol (PEG), and the authors assessed 139 published papers correctly describing PEG-induced dehydration rather than drought in various studied plant species. The authors argued for the use of much clearer terminology in experiments using PEG that strictly limit discussion to “PEG-induced”, “simulated” or “mimicked” dehydration, and osmotic or water stresses, with the best option being “PEG-induced dehydration”. Five special cases are presented, with experiments in both soil/substrate and hydroponics in diverse plants species, including cotton, *Medicago truncatula*, wild barley, maize, and soybean. The authors concluded that the physiological parameters and gene expression profiles differed between plants grown in containers with drying soil and those subjected to dehydration using PEG-induced stress in hydroponics [3].

Plants of bread wheat cultivar Leningradskaya 6 were grown in Hoagland nutrient solution with and without 20% polyethylene glycol-6000 (PEG-6000). This treatment simulated plant dehydration with an osmotic potential of −0.75 MPa compared to controls. Proteomics analyses revealed 497 and 157 differentially abundant protein groups in the leaves and roots, respectively. Oxidative- and heat-stress-responsive proteins were identified in the leaves of the PEG-treated plants, whereas dehydration-related proteins were found in the roots of the treated plants. The authors analysed extracellular root peptides in plants after PEG-induced dehydration, leading to the identification of 16 small secreted peptides and peptides derived from proteins involved in cell wall catabolism, intercellular signaling, and stress response. The results help with better understanding the mechanisms of wheat plant adaptation to dehydration for future application in agriculture [4].

The pod dehiscence (PD) trait was studied in 170 accessions of soybean with diverse phenotypes. A genomics approach was used employing Diversity Arrays Technology (DArT) analysis covering the entire soybean genome with 60K DArT markers, including both Silico-DArT and DArT SNPs. A genome-wide association study (GWAS) identified 14 stable QTLs corresponding to PD- and pod-related traits. Two of the most stable and important genes for PD with known functions were identified from the QTLs, encoding a DNA-binding bromodomain-containing protein and a C2H2 zinc finger protein. Genomic prediction was performed helping to select PD-resistant and -sensitive soybean genotypes with estimated breeding values. The authors concluded that the identified putative candidate genes can be used to improve PD resistance in soybean genotypes using marker-assisted selection and genomic prediction [5].

An in silico analysis of genome assemblies from 34 *Oryza* species, including cultivated and wild rice from available databases, was carried out targeting calcium-dependent protein kinases (CPKs) and other CPK-related kinases. Evolutionary genomics and comparative pan-genome analysis revealed distinct duplication types among the studied groups of CPK genes. Alternative splicing was identified in the transcriptome analysis as a potential mechanism contributing to the diversity of CPK protein domains. Expression studies of CPKs in multiple species of the *Oryza* genus resulted in comprehensive data provided as an atlas for comparison. It would be interesting and important to confirm the presented computer analysis with results from future experiments obtained from plants from various rice species in molecular laboratories [6].

An integrative meta-analysis of publicly available transcriptomic databases was carried out for comparative and evolutionary studies of potato and tomato, two of the most important species from the *Solanum* genus, in response to drought and heat stresses. A robust and reproducible set of stress-responsive genes was generated with reduced experiment-specific variability. Species-specific heat-responsive expression profiles were observed in tomato plants, whereas potato plants showed wide transcriptional activities under drought stress. The potato genomes exhibited lineage-specific gene duplications compared to tomato, suggesting that the duplication event in potato was relatively recent and contributed to stress adaptation. The authors concluded that the results of their meta-analysis provide an integrated view of the molecular and evolutionary mechanisms underlying drought and heat stress responses in two major Solanaceae crops [7].

Zinc is an essential micronutrient that serves as a cofactor for numerous plant enzymes. However, an overaccumulation of zinc in soils can lead to zinc stress, disrupting the normal physiological and metabolic processes of plants. In separate studies, the metabolome and proteome responses of *Amaranthus caudatus* plants to zinc stress were investigated. These studies independently highlighted organ-specific shifts in the metabolite and protein profiles of *A. caudatus* in response to the zinc stress, with the roots being the most metabolically active organ, followed by young leaves and then mature leaves. The authors attributed the high metabolic activity of the roots under zinc stress to the organ’s direct role in zinc uptake and detoxification [8,9].

In the metabolomics study, low-molecular-weight metabolite analysis resulted in the detection of 419 derivatives in the samples, of which 144 could be putatively annotated. Most of the upregulated metabolites in both the leaves and roots were sugars and organic acids, with putative roles in osmotic regulation, oxidative protection, and Zn^2+^ complexation, which the authors highlighted as the probable key feature in the metabolic responses of *A. caudatus* to Zn^2+^ stress. A follow-up proteomic investigation of zinc-stressed *A. caudatus* roots and leaves was conducted using liquid chromatography–mass spectrometry (LC-MS). Overall, the authors highlighted the importance of redox homeostasis, protein synthesis, repair/folding, and degradation systems in *A. caudatus* plants under zinc stress [8,9].

A multi-omics approach integrating physiological, ultrastructural, transcriptomic, and lipidomic analyses was used to investigate the mechanisms of desiccation tolerance and senescence in the resurrection plant *Eragrostis nindensis* [10]. Non-senescent leaf tissue showed remarkable ultrastructural reorganisation, including chloroplasts with disassembled thylakoids, increased vacuolation, and densely accumulating lipid droplets as a survival mechanism under dehydration and desiccation. These features reversed upon tissue rehydration, while senescent leaf tissue cells collapsed and failed to recover. A large-scale transcriptomic analysis of the two tissue types under a range of dehydration levels from full hydration to the air-dry desiccation state further revealed a dynamic regulatory program for desiccation tolerance in *E. nindensis*. For example, genes related to seed-protective functions, autophagy, and stress-responsive pathways were upregulated in non-senescent leaves during dehydration. In contrast, photosynthesis, metabolism, and carbon fixation genes were largely downregulated under dehydration stress, which the authors attributed to the need for a metabolic shutdown and the suppression of energy-intensive processes. Overall, the study expands our understanding of the complex mechanisms of desiccation tolerance in resurrection plants, which include cellular ultrastructural modifications, maintenance of membrane and organelle integrity by lipid droplets, a complex transcriptional regulatory program, and metabolic arrest [10].

A genome-wide identification, classification, and expression study was systematically conducted of the bread wheat *Respiratory burst oxidase homologs* (*TaRboh*) gene family. In silico analysis identified 39 *TaRboh* genes, together with their chromosomal distribution patterns and gene structures. The subsequent characterisation of their *cis* elements suggested that *TaRbohs* may be involved in diverse functions, including phytohormone response, biotic and abiotic stress response, and plant growth. Quantitative expression profiling of nine candidate *TaRboh* genes under 25% PEG-6000-induced dehydration mimicking drought stress, heat, and their combined stress also revealed distinct levels of the responsiveness of the genes to the imposed stresses, thus validating their roles in drought and heat responses. Overall, the study lays a foundation for future molecular investigations into the role of *TaRboh* genes in wheat responses to drought, heat, and these stresses combined [11].

Another study evaluated the effects of chloramphenicol, a broad-spectrum antibiotic, on the germination and early seedling establishment of rice cultivar Khao Dawk Mali L 105, commonly known as Thai jasmine rice [12]. The authors rationalised that chloramphenicol persists in agricultural soils through wastewater contamination and affects growth processes in various plant species, including rice. The results showed that chloramphenicol treatment did not affect germination relative to the control but negatively affected early seedling establishment, with pronounced decreases in seedling vigor across antibiotic concentrations. A phosphoproteomic analysis using LC-MS/MS also revealed stage-specific phosphoproteome changes in 3-day- and 6-day-old geminated rice seeds in response to 15 μg/mL chloramphenicol treatment. These chloramphenicol-responsive phosphoproteins were putatively involved in metabolic- and cytokinesis-related processes during germination and hormonal signaling pathways, stress response, DNA replication, and metabolism during early seedling establishment. The authors concluded that chloramphenicol affects rice seed germination and physiological processes and suggested that future studies should compare the antibiotic’s effects across different rice cultivars to expand our knowledge of plant stress responses [12].

Recent advances in crop functional genomics were discussed for their potential applications in developing stress-tolerant crops for improved global food security. The collective importance of omics-based plant research for identifying stress-responsive genes, proteins, metabolites, and/or metabolic pathways were highlighted as critical for stress tolerance. Genome editing technologies and their applications in plant stress biology were also reviewed, and the authors emphasised the need to critically evaluate the ethical and regulatory elements of these technologies to ensure their responsible and safe use. Potential challenges involved in the translation of laboratory-generated omics data to field studies were especially highlighted [13].

In the context of global warming and the onset of climate change, abiotic stresses are likely to become more intense, resulting in a decline in crop production and increasing disruptions of plant ecology. Molecular tools such as genomics, metabolomics, proteomics, other omics together with multi-omics can improve our understanding of plant responses to such stresses. The identified stress-responsive genes, proteins, and metabolites could help elucidate the biological mechanisms underlying plant tolerance to abiotic stresses. We hope that this Special Issue provides a significant contribution to this process.
